# Skeletal muscle atrophy in sedentary Zucker obese rats is not caused by calpain-mediated muscle damage or lipid peroxidation induced by oxidative stress

**DOI:** 10.1186/s12952-014-0019-z

**Published:** 2014-12-30

**Authors:** Nancy Pompeani, Emma Rybalka, Heidy Latchman, Robyn M Murphy, Kevin Croft, Alan Hayes

**Affiliations:** Centre for Chronic Disease, College of Health and Biomedicine, Victoria University, Melbourne, Australia; Institute of Sport, Exercise and Active Living (ISEAL), Victoria University, Melbourne, Australia; Department of Zoology, La Trobe University, Melbourne, Australia; School of Medicine and Pharmacology, University of Western Australia, Perth, Australia

## Abstract

**Background:**

Skeletal muscle undergoes significant atrophy in Type 2 diabetic patients and animal models. We aimed to determine if atrophy of Zucker rat skeletal muscle was due to the activation of intracellular damage pathways induced by excess reactive oxygen species production (specifically those associated with the peroxidation of lipid membranes) and calpain activity. 14 week old obese Zucker rats and littermate lean controls were injected with 1% Evan’s Blue Dye. Animals were anaesthetised and extensor digitorum longus and soleus muscles were dissected, snap frozen and analysed for ROS-mediated F_2_-isoprostane production and calpain activation/autolysis. Contralateral muscles were histologically analysed for markers of muscle membrane permeability and atrophy.

**Results:**

Muscle mass was lower in extensor digitorum longus and soleus of obese compared with lean animals, concomitant with reduced fibre area. Muscles from obese rats had a higher proportional area of Evan’s Blue Dye fluorescence, albeit this was localised to the interstitium/external sarcolemma. There were no differences in F_2_-isoprostane production when expressed relative to arachidonic acid content, which was lower in the obese EDL and soleus muscles. There were no differences in the activation of either μ-calpain or calpain-3.

**Conclusions:**

This study highlights that atrophy of Zucker rat skeletal muscle is not related to sarcolemmal damage, sustained hyperactivation of the calpain proteases or excessive lipid peroxidation. As such, establishing the correct pathways involved in atrophy is highly important so as to develop more specific treatment options that target the underlying cause. This study has eliminated two of the potential pathways theorised to be responsible.

## Background

Type 2 Diabetes (T2D) is a chronic lifestyle disease characterised by high plasma free fatty acids (FFAs), hyperglycaemia, hyperinsulinaemia and insulin resistance; and which effects multiple organ systems [1]. Notably, the skeletal musculature undergoes significant atrophy [2], which has further adverse impacts on disease progression. Whilst protein synthesis and degradation imbalance likely accounts for this atrophy [3,4], the triggers that lead to changes in the activation of these pathways in T2D and hence alterations in protein turnover are still controversial. Several catalytic pathways have been implicated in atrophic cellular protein degradation including the autophagosome-lysosomal, ubiquitin proteasome, Caspase and Ca^2+^-dependent calpain pathways [5-7]. While a role for the ubiquitin proteasome [8] and autophagosome-lysosomal [9,10] pathways have been established in T2D-related skeletal muscle atrophy, much less is known about the Ca^2+^−dependent calpain system. Indeed, a potential mechanism that may contribute to T2D induced muscle degradation is via increased susceptibility of the skeletal musculature to damage – in particular, via the flow on effects of Ca^2+^ dysregulation, calpain activation and sustained free radical production, both of which are common features of pathological muscle wasting in a variety of diseases [11].

The calpains are a family of Ca^2+^-dependent cysteine proteases – skeletal muscle fibres contain both the ubiquitous isoforms μ-calpain, m-calpain, and calpain-10, as well as the muscle-specific form, calpain-3 [12]. Those calpains activated within a physiologically relevant [Ca^2+^] range are calpain-3 and μ-calpain [13,14]. Calpain 3 plays a role in remodelling and maintaining normal sarcomeric structures, whereas μ-calpain is associated with dismantling sarcomeric structures; and a balance in their activities is important for skeletal muscle integrity (see review [15]). Intracellular Ca^2+^ concentrations above resting cytosolic levels cause autolysis of μ-Calpain and Calpain-3, thus increasing their proteolytic activity [13,16]. Over-activation of calpains due to Ca^2+^ overload has been implicated in many pathological conditions including, Parkinson’s disease and muscular dystrophy [17,18]. As there is substantial evidence suggesting dysregulation of intracellular Ca^2+^ homeostasis in T2D (for review see [19]), it is possible that calpains are being over-activated by excessive intracellular Ca^2+^ accumulation in this disease. Thus far, calpain activity has not been investigated for a role in T2D-associated atrophy.

Much like calcium dysregulation induces damage-associated muscle atrophy, increases in FFA content can lead to excessive production of reactive oxygen species (ROS) and reduced antioxidant defences [20] resulting in damage to proteins, lipids and nucleic acids [21]. With respect to T2D, much of the existing literature on heightened ROS production has been restricted to the mitochondria [22-24] and the impact on whole muscle has not been addressed. High FFA content is also accompanied by changes in the lipid profile of cells which affects membrane integrity and fluidity, as well as leaving membranes more susceptible to ROS-induced lipid peroxidation further impairing their structural integrity [25,26]. Free radical peroxidation of arachidonic acid, a component of cell membranes, forms a prostaglandin-like end product known as the F_2_-isoprostanes. Mass spectrometry assessment of F_2_-isoprostanes is regarded as the gold standard biomarker of oxidative stress (for review see [27]) and plasma F_2_-isoprostanes are significantly higher in both diabetic compared to non-diabetic patients [28] and in obese versus lean diabetic Zucker rats [29] T2D. F_2_-isoprostanes is measurable in skeletal muscle [30], however no study to date has investigated F_2_-isoprostane production in T2D skeletal muscle, and the influence of increased oxidative stress on atrophy and fibre morphology in this disease.

Evans Blue Dye (EBD) has been established as a useful tool to determine cell membrane permeability and can be assessed using different techniques including red auto-fluorescence in tissue sections using fluorescence microscopy [31]. EBD binds to plasma albumin [32] and has been used to identify damaged skeletal muscle fibres which results in them becoming permeable to albumin [31]. As such, EBD would be a useful way to determine membrane permeability and to identify potentially damaged myofibres in T2D – especially given the dysregulation of calcium homeostasis and potential for calpain activation, as well as the effects of ROS and isoprostane production on cell integrity.

Therefore, the aim of this study was to determine if the skeletal muscle atrophy associated with obesity and insulin-resistance in the obese rat animal model of the disease is a result of enhanced membrane fragility mediated by excess ROS and/or increased fibre damage due to excess calpain activity, using EBD fluorescence as a marker. We hypothesised that: (1) the sarcolemma of obese Zucker skeletal muscle would be more permeable to the extracellular fluid (as evidenced by albumin-conjugated EBD fluorescence within myofibres) and show evidence of ROS-induced lipid peroxidation (as determined by F2 isoprostane production); and (2) that increased sarcolemmal permeability would result in hyperactivated calpains. Herein, we confirm hyperinsulinaemia, hyperglycaemia, reduced insulin sensitivity and skeletal muscle atrophy in the T2D obese Zucker rat, but importantly determine that this atrophy is not caused by sarcolemmal damage, elevated lipid peroxidation and degradative calpain activity.

## Results

### Weight & metabolic parameters

**Figure 1:**
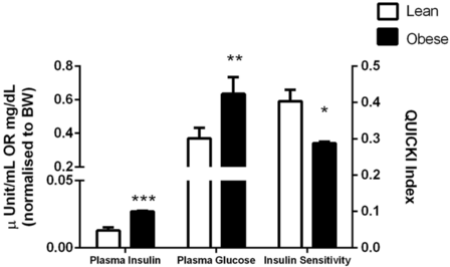
**Plasma insulin and glucose concentration (normalised to body weight) and relative insulin sensitivity as determined by the quantitative insulin sensitivity check index (QUICKI) in lean versus obese Zucker rats (n = 7).** ***p < 0.001; **p < 0.005; *p < 0.05 Lean versus Obese Zucker.

As expected, obese Zucker rats had significantly higher body weights (569.0 g ± 10.8 g versus 369.7 g ± 8.0 g than lean controls; p < 0.01). We have also confirmed hyperglycemia and hyperinsulinemia in the obese Zucker model comparative to lean controls, with plasma glucose and insulin concentrations of 271.2 ± 36.2 mg/dL versus 110.4 ± 18.2 mg/dL (p < 0.005) and 11.8 ± 0.3 μUnits/mL versus 3.8 ± 0.7 μUnits/mL (p < 0.001), respectively (Figure 1). Insulin resistance as determined by the QUICKI method, was also confirmed in the obese Zucker model with an index of 0.29 ± 0.004 compared to 0.40 ± 0.03 in lean controls (p < 0.05; Figure 1).

### Muscle atrophy

**Figure 2:**
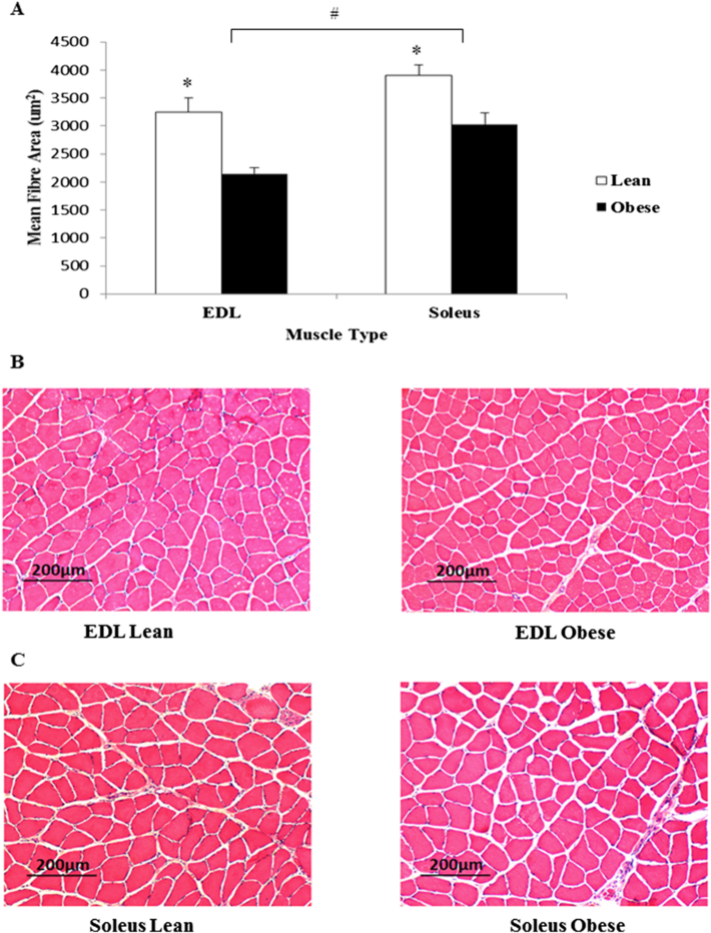
**Skeletal muscle histology. (A)** Average fibre area of skeletal muscle tissues (µm^2^). H & E stained skeletal muscle sections of EDL **(B)** and soleus **(C)** of obese and lean rats. Magnification ×100. *p < 0.01 vs obese. ^#^p < 0.01 vs EDL. n = 14 for each group.

Despite their higher body weights, obese rats displayed significantly lower skeletal muscle weights (0.132 g ± 0.003 g versus 0.167 g ± 0.004 g for lean control EDL and 0.160 g ± 0.007 g versus 0.184 g ± 0.005 g for lean control soleus; p < 0.01) compared to lean rats. The lower muscle weights in the obese rats corresponded with a 35% smaller mean fibre area in EDL (p < 0.01) and 23% smaller fibre area in soleus (p < 0.01) compared to lean controls (Figure 2A-C). Furthermore, fast-twitch EDL fibres were smaller than the slow-twitch soleus muscle fibres (p < 0.01) irrespective of animal group.

### Muscle membrane permeability and damage

**Figure 3:**
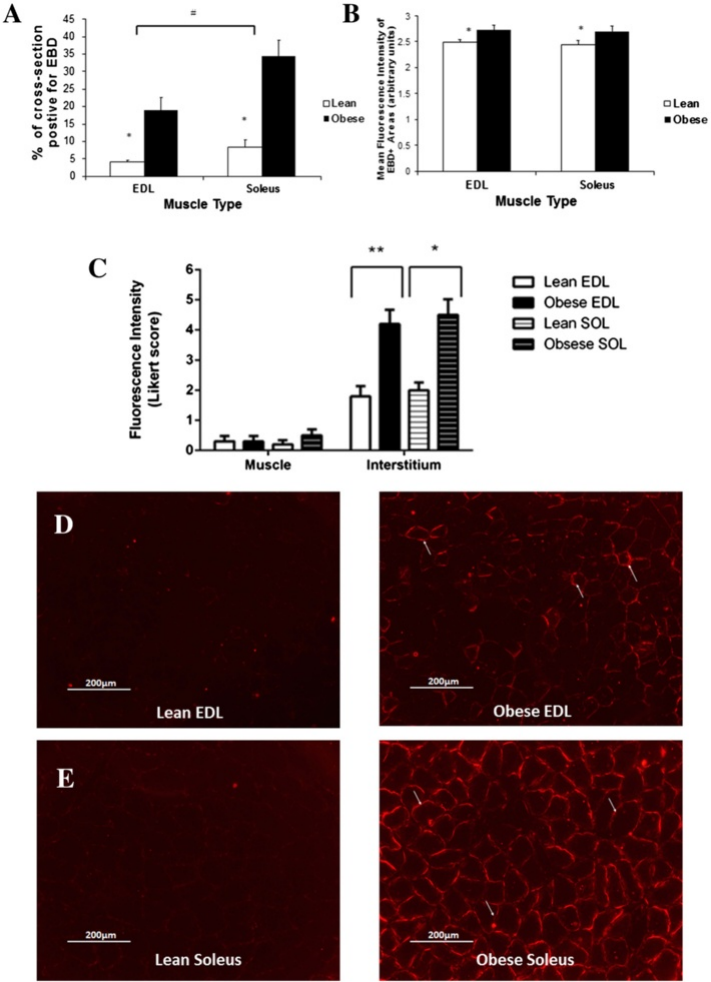
**EBD staining in muscle fibres of Zucker rats.** Proportion (%) of cross-sectional area that is EBD+ **(A)**. Average Fluorescence intensity (arbitrary units) of EBD+ areas **(B)**. Mean strength of EBD signal in myofibres and the interstitium as scored by a semi-quantitative Likert scale **(C)**. Muscle sections of EDL **(D)**, and Soleus **(E)** of obese and lean rats showing EBD accumulation around the skeletal muscle membranes of obese rats (solid arrows), with minimal penetration of the dye in lean rats. Magnification ×100. *p < 0.05 vs obese. ^#^p < 0.05 vs EDL. n = 14 for each animal group.

Obese rats displayed a ~4-fold increase in the % of EBD+ to total cross sectional area in both EDL and soleus sections, compared to lean rats (Figure 3A). In addition, the EBD+ cross sectional area was significantly greater in soleus compared to EDL (p < 0.05). Of the fibre area that stained EBD+, fluorescence intensity (arbitrary fluorescence units) was significantly higher in obese compared to lean rats for both EDL and soleus (p < 0.05) (Figure 3B). The muscle sections from obese rats clearly showed, however, that while exhibiting a higher proportion of EBD fluorescence compared to lean controls, the dye is localised to the interstitium and not penetrating the sarcolemma (as indicated by the solid arrows Figure 3D and E) . As such, the location (intramyofibre or interstitium) of EBD fluorescence within the total muscle section was semi-quantified using a seven-point Likert scale where 0 indicates no signal and 6 indicates very strong signal (Figure 3C). There was no difference in the intramyofibre EBD fluorescence intensity between lean or obese muscle, nor was there any difference between EDL and soleus (p > 0.05). However, the EBD fluorescence intensity was significantly greater in the interstitium of obese compared to lean EDL (p < 0.001) and soleus (p < 0.005).

### ROS-induced F_2_-isoprostane production

**Figure 4:**
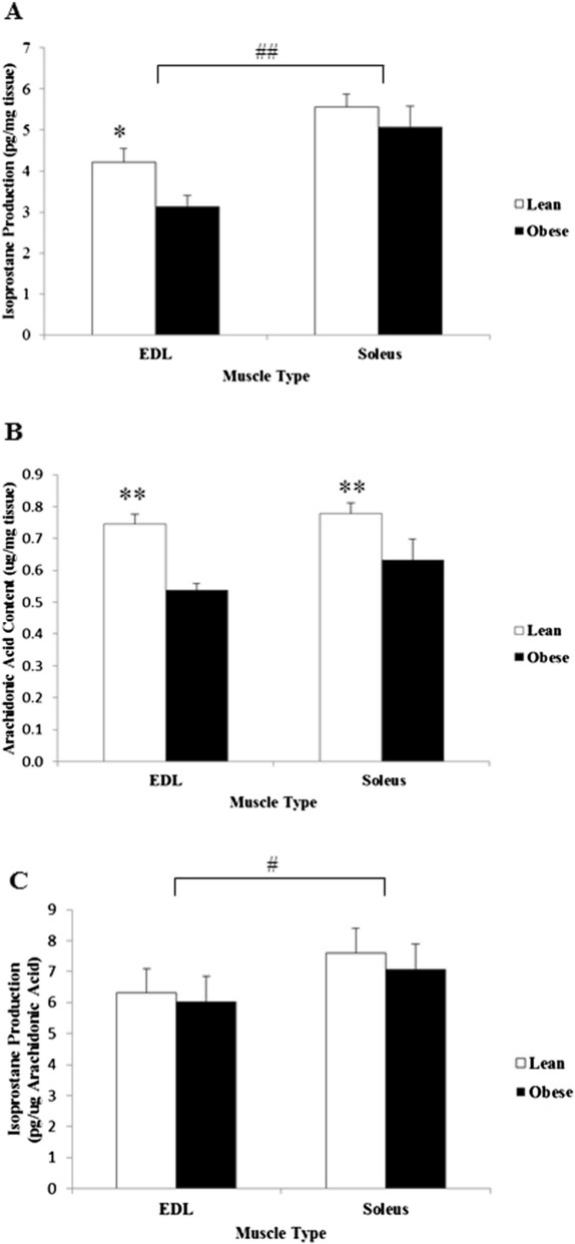
**Isoprostane and arachidonic acid content of Zucker skeletal muscle. (A)** Total muscle F2-isoprostane production per md tissue. **(B)** Total muscle arachidonic acid content. **(C)** F2-isoprostane production corrected for arachidonic acid content. *p < 0.05 vs obese. **p < 0.01 vs obese. ^#^p < 0.05 vs EDL. ^##^p < 0.01 vs EDL. n = 13 for each group.

F_2_-isoprostane production was measured in EDL and soleus muscles. Lean Zucker rats contained 27% more F_2_-isoprostanes compared to obese littermates in EDL (p < 0.05) (Figure 4A), and a similar trend in soleus (p = 0.07). However the lower total arachidonic acid content in both EDL (p < 0.01) and soleus (p < 0.05) muscles in obese compared with lean samples (Figure 4B) indicated comparable F_2_-isoprostane production when corrected for arachidonic acid content (Figure 4C). Soleus muscle produced significantly more F_2_-isoprostanes compared to EDL (p < 0.05) for both groups of animals.

### Calpain analyses

**Figure 5:**
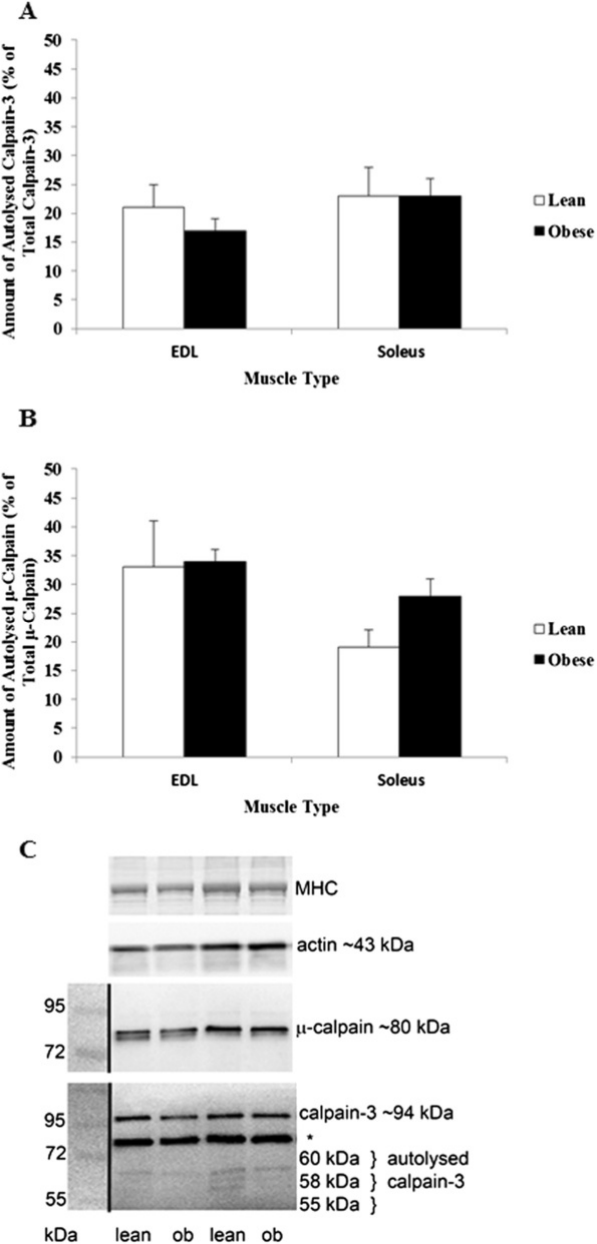
**Calpain activity of Zucker skeletal muscle.** Percentage of activated Calpain-3 **(A)** and µ-Calpain **(B)**. Western blots show either μ-Calpain or Calpain-3 in obese and lean rats **(C)**, with molecular mass markers, taken as a white light image prior to chemiluminescence and then images superimposed, indicated on the left. n = 6 for all groups. *indicates the non-specific band seen in rodent skeletal muscle with this antibody.

To determine if activation of Ca^2+^-mediated proteolytic pathways are a mechanism involved in the muscle atrophy detected in obese Zucker rats, both μ-calpain and calpain-3 autolysis were measured in EDL and soleus (Figure 5). As shown by Western blot analyses (Figure 4B), neither μ-calpain nor calpain-3 autolysis, and hence activation, were increased in obese rats compared to lean rats in either EDL or soleus muscle.

## Discussion

The obese Zucker rat is an established model of T2D mellitus and displays the hallmark biochemical characteristics of the disease including hyperinsulinemia, hyperglycemia and hyperlipidemia, in addition to insulin resistance, as confirmed in this study and by others [33]. At the skeletal muscular level, atrophy is a well-documented consequence of T2D [34,35]. This has been confirmed in the current study, with a significant reduction in the muscle weights of obese Zucker rats compared to lean controls, accompanied by decreased fibre area in both fast- and slow-twitch muscles. Muscular atrophy was observed despite significantly increased body weights in the obese group, in which the increased load-bearing of the musculature should theoretically induce compensatory hypertrophic adaptation to support the body weight. We have investigated the hypothesis that diabetes-associated muscular atrophy is resultant of an increased susceptibility to myocellular damage – specifically due to increased ROS-activated lipid peroxidation of the sarcolemma, permeability of the sarcolemma to the extracellular fluid and hyperactivation of the cytosolic protease calpain. This study highlights that skeletal muscle atrophy of fast and slow twitch fibres from the obese Zucker rat is not directly associated with heightened protein degradation due to physiological [Ca^2+^]-range calpain activation, nor is it resultant of ROS-mediated lipid peroxidation.

Impaired Ca^2+^ regulation has been reported to be an influencing factor in the impaired muscle function and morphology observed in T2D. Given this, [Ca^2+^]-activated calpain autolysis was thought to be a likely mechanism inducing the atrophy in obese muscle. While the direct quantification of intracellular [Ca^2+^] and the Ca^2+^-handling capacity of Zucker skeletal muscle is outside the scope of this study, we have confirmed that any Ca^2+^ dysregulation present is insufficient to induce autolysis of calpain-3 and/or μ-calpain, both of which activate in the presence of heightened [Ca^2+^] within physiological levels. It has been documented that for calpain-3 to become activated, [Ca^2+^]_i_ of 200 nM for at least 60 min is required [13], whereas for u-calpain, 3-50 uM of Ca^2+^ is required [16]. Our data suggests that neither of these [Ca^2+^]_i_ ranges are being maintained for any sufficient period of time to induce sustained calpain-activation and protein degradation to warrant the observed muscle atrophy.

A common cause of persistently elevated [Ca^2+^]_i_, as observed in many muscle pathologies, is sarcolemmal instability which results in microtears within the phospholipid structure and/or hyperactive stretch-induced leak channel activity [36,37]. Using EBD as a marker of muscle damage, we have demonstrated that sarcolemmal integrity is maintained in skeletal muscle from obese rats, suggesting that (1) hyperglycemia and/or hyperinsulinemia do not directly cause instability and/or increased permeability of the sarcolemma; and (2) that muscle damage is not a cause of the observed atrophy. When the sarcolemma is porous, albumin-conjugated EBD can freely move into the muscle where it becomes trapped, resulting in muscle fibres that fluoresce red when viewed microscopically. While this study has demonstrated a higher percentage of EBD fluorescence in skeletal muscle cross-sections of obese rats, dye accumulation was interestingly confined to the external membrane/interstitium rather than being evenly distributed throughout the sarcoplasm as is evidenced in EBD+ sections of damaged skeletal muscle from the *mdx* mouse model of Duchenne Muscular Dystrophy [32] – a severe muscle wasting disease of which a feature is sarcolemmal instability and hyperactivation of Ca^2+^/calpain-induced damage pathways. This demonstrates comparable sarcolemmal integrity between sedentary obese Zucker rats and lean controls, with no obvious signs of elevated muscle damage to the diabetic condition. To our knowledge, accumulation of EBD-conjugated albumin at the extracellular sarcolemmal surface has not been reported previously in diabetic skeletal muscle. We speculate that this is reflective of increased extravasation of EBD-bound albumin from associated capillaries [38] and subsequent binding of albumin to membrane glycoproteins [39], which are notably overexpressed on the extracellular surface of membranes in response to chronic exposure to a hyperglycaemic environment [40]. Excessive albumin accumulation demonstrably promotes modification of the size and compilation of the interstitium [41], reduces membrane fluidity [42] and binds Ca^2+^_e_ [43]. While such an effect may serve as a protective mechanism to hyperglycaemia, how this would impact upon normal skeletal muscle preservation and function is currently unknown, although impaired nutrient diffusion and reduced nutrient availability to ATP synthesis could be a likely outcome.

ROS-induced F_2_-isoprostane production (measured as a marker of lipid peroxidation) was hypothesised as another potential mechanism responsible for the atrophy found in the obese Zucker model of diabetes. Previous research has demonstrated increased F_2_-isoprostanes in plasma of patients with T2D [28]. However, our results indicate that F_2_-isoprostane production was comparable in both EDL and soleus muscle from obese compared to lean rats. Isoprostane formation was significantly lower (data not shown) in muscle from obese rats compared to lean controls, however, so too was total arachidonic acid content (data not shown). Thus, when corrected for total arachidonic acid content, F2-isprostane production was comparable between obese Zucker and control skeletal muscle. This correction is essential given the reliance on arachidonic acid availability for isoprostane production. Whether this decrease in arachidonic acid content (and thus isoprostane production) is reflective of prior sarcolemmal damage, or simply less capacity for production, is unknown. As albumin has a tendency to bind to, and stimulate, the release of arachidonic acid from the membrane [42] and our EBD data demonstrates EBD-conjugated albumin accumulation in the interstitial space proximal to the extracellular sarcolemmal surface, we speculate that albumin is having a direct effect on the arachidonic acid content of the sarcolemma in obese Zucker muscle. Low membrane arachidonic acid content, as has been demonstrated in the current study, also reportedly reduces membrane fluidity [42]. How changes to sarcolemmal fluidity effect normal skeletal muscle preservation and function is currently unknown. A notable limitation of our study is that we have included only F2-isoprostane measurement as a marker of lipid peroxidation – future research would benefit from parallel analysis of lipid peroxidation byproducts such as malondialdehyde (MDA) and 4-hydroxynonenal (4-HNE) to enable complete assessment of membrane lipid damage.

Another interesting observation was that the predominantly slow-twitch soleus muscle displayed a higher rate of F_2_-isoprostane production than predominantly fast-twitch EDL muscle. Although more glycolytic in nature, Type II fibres are demonstrably more susceptible to oxidative damage than Type I fibres. Since our soleus muscle sections also had higher albumin-conjugated EBD fluorescence in the interstitium (likely due to their higher capillary density [44]) comparative to glycolytic EDL sections, we speculate that this albumin is actively binding to arachidonic acid and releasing it from the sarcolemma [45]. This highlights that increased vascular permeability, and in particular extravasation of albumin into the interstitium, renders oxidative muscle fibres more susceptible to membrane changes that may subsequently affect muscle function.

Skeletal muscle mass is highly regulated by a variety of molecular pathways that promote or inhibit protein synthesis and degradation. While our data importantly demonstrates that atrophy induced by the diabetic phenotype is not linked to sarcolemmal porosity and the ensuing protein degradative pathways controlled by [Ca^2+^]_i_-induced calpains, there are many other signaling mechanisms that may be contributing to the atrogenic environment. Indeed, the protein synthetic phosphatidylinositol 3-kinase (PI3K)/phosphorylated Akt (pAkt) [46] and the protein degradative ubiquitin-proteasome proteolytic pathway [47] have both been linked to T2D. The triggers that lead to activation of these pathways and subsequent alterations in protein turnover in T2D are still controversial, however our data alongside others [4] suggests that inhibition of protein synthesis may be the overriding regulator. In addition to impaired glucose uptake into skeletal muscle, insulin resistance also reduces amino acid uptake [48] which may explain the slower skeletal muscle protein synthesis rates observed in this disease [4]. Paturi et al. [49] have confirmed reduced mTOR activation in skeletal muscle of obese rats, which highlights that specifically targeting protein synthesis may be an effective way of reversing/preventing the muscle atrophy observed in diabetic skeletal muscles.

## Conclusions

We have provided further evidence that obese Zucker rat skeletal muscles are atrophic. The mechanisms are demonstrably not related to sarcolemmal damage, protein degradation induced by heightened calpain activation or ROS-mediated lipid peroxidation. Additionally, we have shown accumulation of albumin (as evidenced by EBD fluorescence) on the extracellular surface of the sarcolemma and interstitium which may be indicative of capillary macromolecule extravasation – what effect this has on skeletal muscle function and mass preservation is unknown.

## Methods

### Animals

A total of 42 rats were used in this study: 14-week-old male obese Zucker and age-matched, non-diabetic lean Zucker littermates (n = 7 for T2D biomarker experiments and n = 14 for muscle histology, calpain and F_2_-isoprostane) served as the experimental and control groups, respectively (Flinders University, Adelaide, Australia). Rats of the same phenotype were housed in pairs and allowed access to food and water *ad libitum*. All experiments were approved by the Victoria University Animal Ethics Experimentation Committee and conformed to the Australian Code of Practice for the Care and Use of Animals for Scientific Purposes.

### Biochemical measures

While obese Zucker rats demonstrably develop insulin-resistance by 14 weeks of age [33], we have quantified plasma glucose and insulin concentrations and calculated insulin sensitivity to confirm T2D in our colony. On a separate group of fasted (overnight) and anaethetised rats (n = 7 for obese and lean groups), blood samples were collected via cardiac puncture into eppendorf tubes and centrifuged (10 min at 13,300 RPM). Plasma was decanted and immediately analysed for plasma glucose using a Yellow Springs analyser (Yellow Springs Instruments, Ohio, USA). Insulin concentration was determined in 10 μl of plasma by ELISA according to the protocol (Rat/Mouse Insulin ELISA Kit) provided by the manufacturer (Linco Research). Using the derived plasma glucose and insulin concentrations, insulin sensitivity was calculated using the quantitative insulin sensitivity check index (QUICKI) method (QUICKI = 1/[log(I_0_) + log(G_0_)]) [50].

### Muscle sampling protocol

Animals were injected with EBD as optimised for skeletal muscle by Hamer et al. [32]. A 1% EBD solution (Ajax Chemicals, 32688) (w/v) in phosphate-buffered saline (PBS, pH 7.5), was filtered through a Millex-GP 0.22 μm filter and stored at 4°C. Twenty four hours prior to muscle sampling, rats were given an intraperitoneal (I.P.) injection of 1% EBD (v/w). After the injection, animals were returned to their cage and allowed access to food and water *ad libitum* for 24 hours to allow optimal uptake of the dye into any leaky myofibres [32].

On the day of muscle sampling, rats were weighed and anaesthetised with Pentobarbitone Sodium (60 mg.kg^−1^ body weight). The Extensor Digitorum Longus (EDL, fast-twitch), and Soleus (slow-twitch) muscles were excised, cleaned of excess fat and connective tissue, including tendons, and weighed on standard laboratory scales. The tissue collected was covered with Tissue-Tek and frozen in isopentane cooled in liquid nitrogen for histological analyses of muscle membrane permeability, damage and atrophy. EDL and soleus muscles from the alternate leg were then removed, and snap frozen in liquid nitrogen for analysis of isoprostane production and calpain activity.

### Muscle membrane permeability, damage and atrophy

Frozen embedded sections were cut (10 μm) at −21°C on a cryostat, dipped in cold acetone (Merck) (−20°C) for 1 min and air dried at room temperature. Sections were then dipped into xylene (Merck) and mounted with DPx (Fluka Biochemika) and a coverslip. Another slide of consecutive serial sections was stained with Haemotoxylin and Eosin (H&E) to determine muscle fibre size. In a single-blinded protocol, 100 fibres per section were manually traced using Analytical Imaging Station (AIS) software and the cross sectional area (μm^2^) of each fibre was determined. The area analysed was chosen by placing the section in the centre of the field of vision at low (40x) magnification, and then counting the fibres in view when changing to the higher (100x) magnification.

EBD sections were visualised by fluorescence microscopy using a N2.1 green wavelength filter set (band pass = 515-560 nm; (low pass = 590 nm). The proportion of cross-sectional area positive for EBD (EBD+) was determined and the fluorescence intensity (relative units) of EBD+ areas was quantified. The location of EBD fluorescence intensity in the frozen sections was assessed semi-quantitatively using a seven-point Likert scale scoring system as per Hamer et al. [32]. The scale, from 0 to 6, was defined as ranging from 0 = no signal; 1 = minimal signal; 2 = weak signal; 3 = good signal; 4 = moderate signal; 5 = strong signal; 6 very strong signal. A score on this scale for EBD red fluorescence was recorded for both myofibre penetration and the interstitium, viewed at a final magnification of 100x. H&E sections were viewed with light microscopy (Zeiss Axiolab, Carl Zeiss GmbH)

### ROS-induced F_2_-isoprostane production

Tissue F_2_-isoprostanes were measured as previously described [51]. F_2_-isoprostanes were detected by electron-capture negative ionization GC-MS after solid-phase extraction and corrected for total arachidonic acid content. Arachidonic acid was measured in lipid extracts from frozen muscle, after conversion to the methyl esters with 2 mL 4% H_2_SO_4_ in methanol (90 C, 10 min). The methyl esters of fatty acids were analysed by gas chromatography as previously described [34]. Heptadecanoic acid (50 μL of stock 1 mg/mL) was used as internal standard.

### Calpain analyses

μ-calpain and calpain-3 autolysis was determined in skeletal muscle from obese (n = 6) and lean (n = 6) rats. Muscle samples were homogenised using 10 volumes of ice-cold extraction buffer, comprising 0.4 M Tris-Cl, pH 6.8, and 25 mM EGTA ([Ca^2+^] < 10 mM). SDS was added to a final concentration of 4%. Muscle homogenates were incubated at 4°C for 20-40 min, an aliquot was kept for protein concentration assay (Quant-iT fluorescence assay, Invitrogen), and the remaining homogenate was diluted 1:5 v/v with extraction buffer. This was added to SDS loading buffer (2:1 v/v) comprising of 0.125 M Tris HCl, 10% glycerol, 4% SDS, 4 M urea, 10% mercaptoethanol, and 0.001% bromophenol blue, pH 6.8 which had been diluted (2:1 v/v) in physiological based solution. Samples were stored at −20°C until analysis. Samples were analysed by Western blotting as previously described [13]. Total protein from muscle samples were separated on an 8% SDS-PAGE gel and transferred to nitrocellulose membranes. The membranes were probed with antibodies against μ-Calpain (1:1,000 mouse monoclonal, Sigma monoclonal, clone 15C10), and calpain-3 (1:200 mouse monoclonal, Novocastra monoclonal 12A2), and goat anti-mouse horseradish peroxidise (HRP) (1:50,000 Bio-Rad) was then added to the membranes. Bands were visualised with West Pico chemiluminescent substrate (Pierce), and densitometry was completed using Quantity One software (Bio-Rad). Once transferred, gels were stained with BioSafe Coomassie blue (Bio-Rad), and myosin heavy chain (MHC) in the post-transferred gel as well as membranes probed with actin (Sigma A-2066) were used as an indicator of sample loaded [16]. Full-length μ-calpain was visualised as an 80 kDa protein, and its activation was confirmed by its autolysis to 78- and 76-kDa proteins. Calpain-3 was observed as a 94-kDa protein with activation confirmed by autolysis to proteins of approximately 60-, 58-, and 55-kDa [14]. Data for the Western blots are presented as the density of the bands corresponding to the autolysed products relative to the density of the total bands representing μ-calpain or calpain-3 for a given sample. This indicated the proportion of μ-calpain or calpain-3 that was autolysed in a particular sample, irrespective of any minor differences in protein loading.

### Data analyses

Results are expressed as means ± Standard Error of Mean (SEM) and compared by two-way ANOVA, with animal strain and muscle type as factors. No significant interactions were detected. In all cases a P < 0.05 was considered statistically significant.
